# A Dual-Module System for Copyright-Free Image Recommendation and Infringement Detection in Educational Materials

**DOI:** 10.3390/jimaging10110277

**Published:** 2024-11-01

**Authors:** Yeongha Kim, Soyeon Kim, Seonghyun Min, Youngung Han, Ohyoung Lee, Wongyum Kim

**Affiliations:** 1 R&D Center, AIDEEP Co., Ltd., 25, 7na-gil Gwacheon-daero, Gwacheon-si 13840, Republic of Korea; yhkim@aideep.ai (Y.K.); shmin@aideep.ai (S.M.); yuhan@aideep.ai (Y.H.); 2 Tekville, 551, Eonju-ro, Gangnam-gu, Seoul 06138, Republic of Korea; 250.lee@tekville.com

**Keywords:** copyright protection, copyright-free, image retrieval, infringement detection

## Abstract

Images are extensively utilized in educational materials due to their efficacy in conveying complex concepts. However, unauthorized use of images frequently results in legal issues related to copyright infringement. To mitigate this problem, we introduce a dual-module system specifically designed for educators. The first module, a copyright infringement detection system, employs deep learning techniques to verify the copyright status of images. It utilizes a Convolutional Variational Autoencoder (CVAE) model to extract significant features from copyrighted images and compares them against user-provided images. If infringement is detected, the second module, an image retrieval system, recommends alternative copyright-free images using a Vision Transformer (ViT)-based hashing model. Evaluation on benchmark datasets demonstrates the system’s effectiveness, achieving a mean Average Precision (mAP) of 0.812 on the Flickr25k dataset. Additionally, a user study involving 65 teachers indicates high satisfaction levels, particularly in addressing copyright concerns and ease of use. Our system significantly aids educators in creating educational materials that comply with copyright regulations.

## 1. Introduction

In today’s digital landscape, images are a fundamental medium for communication, information dissemination, and expression. Their capacity to convey complex information succinctly and clearly makes them invaluable, not only in everyday online interactions but also as educational tools. However, utilizing images without the copyright holder’s permission or using images with unverified copyright status can result in legal complications related to copyright infringement.

In South Korea, copyright law supports educational institutions in publishing copyrighted works in textbooks necessary for educational purposes. According to Article 25, Paragraph 7 of the Copyright Act, the Korea Literary and Artistic Copyright Association (KOLAA), designated by the Minister of Culture, Sports, and Tourism, receives an annual “Statement of Use of Copyrighted Works” and royalties from institutions using copyrighted materials for instructional purposes. KOLAA then distributes these royalties to the respective copyright holders, ensuring they receive fair compensation and protecting their rights. Consequently, teachers can freely use published copyrighted images in their teaching materials without worrying about copyright issues, significantly aiding in improving students’ understanding, maximizing learning outcomes, and enhancing the quality of lessons.

This paper proposes a copyright-free image recommendation system designed to alleviate these concerns. The system helps educators determine whether the images they choose for educational materials may result in copyright infringement. Furthermore, it retrieves similar images from a database of verified copyright-free images when there is a risk of copyright issues with the selected images.

The system is composed of two modules. The copyright infringement detection module determines whether the image provided by the user is a published copyrighted work registered with KOLAA. Using a deep learning-based model, the Convolutional Variational Autoencoder (CVAE) [1], meaningful feature vectors are extracted from the copyrighted images, which are then converted into two types of keys (first and second keys) for faster searching within the database and storage in the database. Subsequently, the image provided by the user is compared with the feature information in the database to determine whether it is a verified copyrighted image that exists in the copyright database.

If the inspected image is likely to result in copyright infringement, the image retrieval module suggests similar images from the copyright-free image database using an image hashing model. By extracting a hash code from the user-provided image and comparing it with vectors in the database, the system effectively recommends similar images. Through this process, users can easily find and actively utilize copyright-free images from the copyright database for educational material creation.

The main contributions of the proposed system are as follows:

- The system integrates copyright infringement detection and similar image retrieval functionalities to help educators create educational materials without infringing on copyrights. This approach prevents copyright issues in advance and enhances the efficiency and safety of educational material creation by recommending copyright-verified images.
- The network structure of the image retrieval model was improved by using the Vision Transformer (ViT) [2] as the backbone network, enhancing the system’s ability to capture global feature information from images. Additionally, a new loss function was introduced to improve the model’s training efficiency and the quality of retrieved images.
- The system’s utility was verified through user satisfaction surveys and performance evaluations of the model. The image hashing model outperformed other hashing models on the Flickr25k [3], CIFAR10 [4] and NUS-WIDE [5] benchmark datasets. Surveys conducted among educators showed high satisfaction with the system and alleviated concerns about copyright infringement.

The structure of this paper Is as follows: In Section 2, Related Work, we review studies related to copyright systems and image retrieval. Section 3, Copyright-free Image Recommendation System, provides an overview of the system, describing the infringement detection module and the image retrieval module. Section 4 analyzes the experimental results of the image retrieval model and the service satisfaction results. In Section 5, Conclusions, the research findings are discussed in-depth and the final conclusion summarizes the main findings and implications of the study.

## 2. Related Work

The protection of digital image copyrights has become an increasingly important issue with the advancement of the information society. In particular, with the proliferation of the internet and social media, there has been a significant increase in the indiscriminate distribution of images and cases of copyright infringement, leading to the proposal of various technical approaches to address this issue. Traditional methods such as digital watermarking, signatures, and hash functions have steadily evolved since their early research stages and have established themselves as fundamental technologies for copyright protection to this day. Ref. [6] proposed a system that uses watermarking technology to protect the copyrights of digital images. This system embeds a watermark containing copyright information into the image and detects it to verify whether copyright infringement has occurred. Subsequently, research was conducted to strengthen copyright protection by utilizing blockchain technology.

Khare et al. [7] proposed a copyright infringement detection system that combines artificial intelligence with blockchain. This system extracts image features and compares them with copyright information stored in the blockchain to determine whether infringement has occurred. Recent studies have suggested methods to enhance copyright protection using the latest technologies, such as artificial intelligence and deep learning. Sun and Zhou [8] proposed a system that compares image similarity using deep perceptual hashing based on hash-centered techniques to detect copyright infringement. Additionally, Kim et al. [9] proposed a framework to accurately handle the manipulation of copyrighted photos. This framework detects the Region of Interest (RoI) in the image, generates binary descriptors from the detected RoI, and compares them with a database to search for similar images.

Previous studies have primarily focused on preventing the replication and infringement of copyrighted images. In contrast, our system aims to verify the copyright status of an image to prevent copyright infringement issues and retrieve copyright-free images that pose no legal concerns. This distinction sets our system apart from existing research, as it emphasizes preemptively addressing copyright issues and providing safe images for educational material creation. To achieve this, we have implemented a deep learning model to effectively perform image infringement detection and retrieval.

One of the recent studies on image copyright verification technology is the autoencoder-based copyright image authentication model proposed by Yang et al. [10]. The CVAE used in this model effectively extracts the essential spatial features of an image by utilizing convolutional filters and enables efficient copyright image authentication by generating and reconstructing latent vectors of the input image using a variational autoencoder structure. It handles various image formats and resolutions, converting high-dimensional data into a lower-dimensional latent space, thus maintaining critical features while accurately assessing image similarity. Zajic et al. [11] proposed an algorithm for content-based image retrieval (CBIR). This methodology involves extracting and processing image features based on color, texture, and shape. In the initial search phase, similar images to the query image are selected based on Euclidean distance. This process is refined iteratively using a Radial Basis Function (RBF)-type neural network to improve the search results, allowing the user to refine and filter the results. TBH [12] introduced a dual bottleneck structure to address the chronic issue of information loss in hashing models. The model adds a binary bottleneck and a continuous bottleneck to the basic autoencoder and utilizes Graph Convolution [13] to learn the correlations between images within a batch. TBH avoids information loss by combining continuous features with the input feature vectors based on the similarity matrix calculated from the binary bottleneck.

Wang et al. [14] proposes a hash center generation method with minimal distance constraints to improve the performance of deep hashing-based image retrieval. This approach ensures sufficient separation between the hash codes of different classes to enhance retrieval accuracy and introduces an optimization technique that guarantees the distance between hash centers, effectively reflecting image similarity and class separation. He and Wei [15] propose a Transformer-Based Deep Hashing (TDH) method aimed at enhancing image retrieval performance by fusing multi-scale features. To achieve this, a hierarchical Transformer structure is employed to simultaneously learn both the global and local features and a Multi-Scale Feature Fusion (MSFF) module is designed to capture richer image information. This approach effectively extracts detailed image information and improves retrieval accuracy. In addition to these approaches, Jose et al. [16] proposed a hash center update strategy that enhances image retrieval performance by optimizing hash center distances. Similarly, Li et al. [17] leveraged pre-trained deep neural networks to improve hashing efficiency, which effectively boosts the retrieval process. Furthermore, Meng et al. [18] introduced an unsupervised deep triplet hashing technique to achieve better feature representation and image retrieval performance, while Meng et al. [19] focused on a dynamic similarity learning approach to optimize hashing for image retrieval tasks. These collective advancements demonstrate the growing impact of deep learning-based hashing methods in achieving accurate and efficient image retrieval.

## 3. Copyright-Free Image Recommendation System

The system is designed to assist educators in creating materials that comply with copyright regulations. In the following sections, we provide a comprehensive overview of the system architecture and explain the functionality of each module in detail.

### 3.1. System Overview

**Figure 1:**
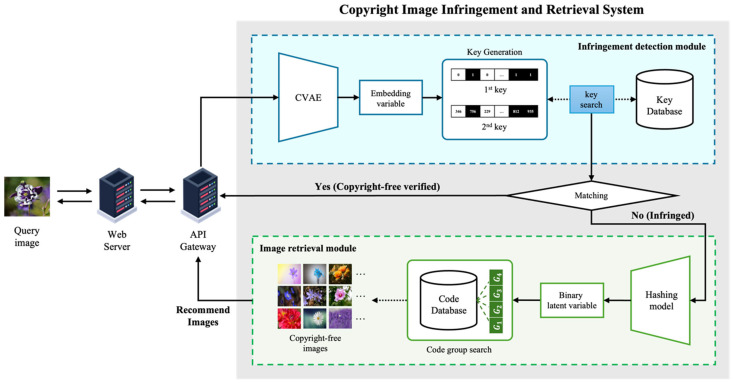
System architecture of the copyright-free image recommendation system.

Figure 1 presents an overview of the copyright-free image recommendation system, which consists of two main components: an image infringement detection module and an image retrieval module.

The image infringement detection module processes the query image uploaded by the user to determine whether it matches any copyrighted images in the database. This module employs a model based on the previously developed CVAE. The uploaded image is passed through the encoder of the pre-trained CVAE to extract its feature information. In a two-step process, unique feature vectors, referred to as the first and second keys, are generated. These keys act as distinctive identifiers that represent the image’s characteristics.

The search is conducted in two stages by comparing these keys with those stored in the copyright database. In the first stage, the system calculates the Hamming distance between the first key of the query image and the key of the images in the database, selecting results that fall below the threshold $T_{1st}$. If a single result is found, it is treated as the final result. If multiple results or no results are found, the system proceeds to the second stage, where it compares the second key using cosine similarity. The result with the highest similarity exceeding the threshold $T_{2nd}$ is selected as the final match. If the final result is determined, the system informs the user that the image is confirmed to be copyrighted. If the thresholds are not met, the system redirects the user to the image retrieval module, which suggests similar images from a copyright-free database.

The image retrieval module uses an improved model of Twin-Bottleneck Hashing (TBH), one of the deep hashing models, to extract feature information from the query image provided by the user and convert it into a binary vector. The search is then performed in the copyright-free image database by calculating the Hamming distance in a manner similar to the first step of the infringement detection process. The results are ranked in ascending order based on the Hamming distance, with the top N images returned to the user. The proposed system is designed based on a web server and API server, allowing users to access and use its functionalities through a web interface.

### 3.2. Infringement Detection Module

**Figure 2:**
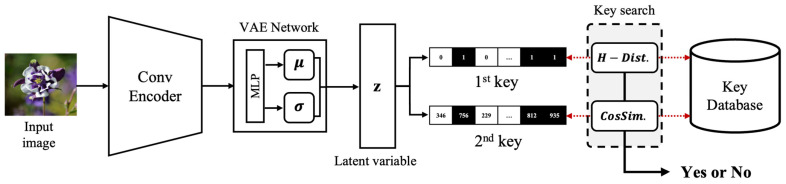
Copyright infringement detection process using CVAE.

The copyright image infringement detection module described in Figure 2 utilizes a deep learning model based on the CVAE to extract features from images and determine whether they are copyrighted by comparing these features with the information stored in the database. The CVAE predicts a probability distribution that accurately represents the given input image through the encoder, while the decoder generates a new image similar to the input image using the estimated probability distribution. In this paper, we adopt the CVAE model proposed by Yang et al. to take advantage of its ability to capture and compress key information from the input image and integrate it into the copyright infringement detection module.

When a query image is provided by the user, the encoder extracts local features through multiple convolutional filters, identifying the key characteristics of the image and compressing them into a lower dimension. More specifically, the input image, with a size of 128 × 128, is passed through five convolutional layers, resulting in a flattened representation of 8192 dimensions. This is then processed through a dense layer, ultimately compressing the representation into a 2048-dimensional feature map. This compressed feature information is then used to predict the probability distributions μ and σ, from which a 64-dimensional embedding vector z is sampled, effectively capturing the unique attributes of the image. The first and second keys generated from the embedding vector z are then compared with the key database to produce the final detection result. The method for generating and matching these keys is explained in detail below.

#### 3.2.1. First Key Generation

The first key, $K^{1st}\in R^{M}$, is generated from the query image through the following process. First, the mean $A^{1st}\in R^{M}$ of the embedding vectors $z\in R^{N,M}$ for all the copyrighted images used during model training is pre-calculated. Here, N represents the total number of images and M is the size of the embedding vector z, which also corresponds to the common key length for both the first key and second key. Then, when a query image is provided by the user, the embedding vector is obtained through the CVAE, and the first key $K^{1st}$ is generated by comparing it with the mean $A^{1st}$ of the embedding vectors of the copyrighted images.

(1) $$K^{1st}=\left\{\begin{array}{c} if\;z_{i}\geq A_{i}^{1st},\;K_{i}^{1st}=1\\ if\;z_{i}<A_{i}^{1st},\;K_{i}^{1st}=0\; \end{array}\right.$$

#### 3.2.2. Second Key Generation

The second key, $K^{2nd}\in R^{M}$, is generated from the embedding vector z by rounding each element of z to the fourth decimal place and then multiplying by 1000 to convert it into an integer, as shown in Equation (2):

(2) $$K^{2nd}=round\left(z,4\right)\times 1000.$$

This process narrows down the values of the embedding vector to a certain number of digits, thereby reducing the data size and computational complexity during subsequent comparison and retrieval processes.

The reason for binarizing and integerizing $K^{1st}$ and $K^{2nd}$ during key generation is to improve system efficiency. If the feature vector composed of real values is used directly to perform searches within the database, it would require significant computational resources and time due to the need to find similar values through matrix multiplication of real vectors. Therefore, by adopting the above key generation method, more rapid and efficient searches can be achieved. Binarized and integerized keys simplify comparison operations, increasing processing speed and enhancing system performance.

#### 3.2.3. Key Search

The generated $K^{1st}$ and $K^{2nd}$ undergo a comparison process with the copyrighted images in the database to verify the copyright status of the query image. The first key $K_{Q}^{1st}$ of the query image is compared with $K_{DB}^{1st}$ of the copyrighted images in the database by calculating the Hamming distance, searching for images in the database that have a Hamming distance less than or equal to $T_{1st}$.

(3) $$\left\{R\in DB\right|\;{H(K}_{Q}^{1st},K_{DB}^{1st})\leq T_{1st}\}\;\in \;R^{1st}.$$

In Equation (3), R represents each key entry in the database, $R^{1st}$ denotes the complete search results from the first key, H is the function that calculates the Hamming distance, and $T_{1st}$ represents the threshold for the first key. If multiple results are returned from the first search, a secondary search is conducted using the second key. The second key $K^{2nd}$ of the query image is compared with $K^{2nd}$ of the filtered images from the first search using cosine similarity. Images with a cosine similarity greater than the second key threshold $T_{2nd}$ are filtered, and the image with the highest cosine similarity is selected as the final search result. This process is expressed in Equations (4) and (5):

(4) $$\left\{R\in R^{1st}\;\right|\;{C(K}_{Q}^{2nd},K_{DB}^{2nd})\;\geq \;T_{2nd}\}\in \;R^{2nd}.$$

(5) $$R^{final}=R^{2nd}[argmax\left(C^{2nd}\right)].$$

In Equation (4), $K_{Q}^{2nd}$ and $K_{DB}^{2nd}$ represent the second key values of the query image and the copyrighted images in the DB, respectively, and is the function that calculates cosine similarity. In Equation (5), $C^{2nd}$ refers to the cosine similarity values calculated during the second key search process. $R^{final}$ represents the final search result with the highest cosine similarity.

In the proposed system, the first threshold $T_{1st}$ is set to 12, and the second threshold $T_{2nd}$ is set to 0.9 to limit the search range. If the first threshold is exceeded or the second threshold is not met, resulting in no final search result, the copyright status of the image cannot be verified. In such cases, the system redirects to the image retrieval module, which suggests similar copyright-free images available in the copyright retrieval DB.

### 3.3. Image Retrieval Module

This chapter explains specific technical approaches to enhance the performance of the image retrieval module. It describes the integration of the Vision Transformer (ViT) backbone network, an image model based on transformers [20], into the existing TBH model and the introduction of a loss function to improve performance through efficient model training. Following this, methods to maximize search efficiency in large-scale databases are introduced, including a Hamming-distance-based search method and search optimization using binary vector code groups.

#### 3.3.1. Deep Hashing Model

**Figure 3:**
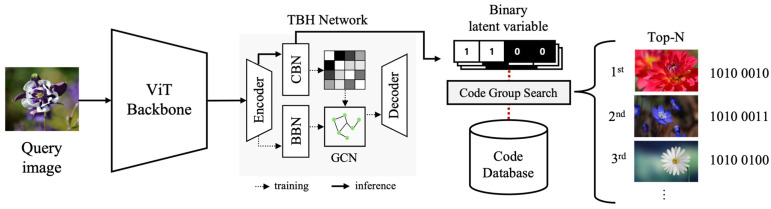
Image retrieval process.

Figure 3 illustrates the structure of the copyright image retrieval module, which performs image retrieval tasks using a deep hashing-based model. By hashing the feature information of images into binary vectors and storing them in a database, similar images can be quickly located among a vast number of images. The TBH we adopted employs an auto-encoding structure with two bottlenecks: a binary bottleneck and a continuous latent variable. The binary bottleneck builds an adaptive similarity graph based on Hamming distance, while the continuous bottleneck adjusts the data through GCN before feeding them into the decoder. The decoder reconstructs the input data, and the reconstruction loss compensates for the encoding quality of the encoder. This model is fully trainable via SGD and overcomes the limitations of static graph problems, generating more discriminative binary codes.

In this study, we made the following improvements to enhance the model’s representational capacity. First, we replaced the AlexNet model, previously used as the backbone of the TBH model, with the ViT model. While CNN-based backbones capture hierarchical features using small filters, they have limitations in considering the overall context of the image. In contrast, the ViT model divides an image into fixed-size patches, linearly embeds each patch, adds positional embeddings, and feeds them into a transformer encoder. The encoder, composed of multi-head self-attention and MLP blocks, models the relationships between patches and integrates information. Additionally, ViT exhibits less image-specific inductive bias than CNNs and shows generalized performance across various image types. These characteristics enable ViT to demonstrate superior performance in large-scale datasets and diverse image recognition tasks.

Next, we accelerated training by directly passing prior knowledge of inter-image relationships from the backbone to the bottleneck in the hashing model. The additional loss function was designed to minimize the mean squared error (MSE) between the adjacency matrices generated at the backbone network and bottleneck. The original TBH model consists of the sum of the autoencoder loss and the discriminator loss, calculated through Equations (6) and (7).

(6) $${\nabla L}_{AE}\approx \frac{1}{B}\sum_{i=1}^{B}E_{b_{i}}\left[\nabla (\frac{1}{2}{\left\|x_{i}-{\hat{x}}_{i}\right\|}^{2}-\lambda \;log\;D_{1}\left(b_{i};\varphi_{1}\right)-\lambda \;log\;D_{2}\left({z^{\prime }}_{i};\varphi_{2}\right))\right],$$

(7) $$L_{D}=\frac{\lambda }{B}\sum_{i=1}^{B}\left(\log d_{1}\left(y_{i}^{\left(b\right)};\varphi_{1}\right)+\log d_{2}\left(y_{i}^{\left(c\right)};\varphi_{2}\right)+\log(1-d_{1}\left(b_{i};\varphi_{1}\right))+\log(1-d_{2}\left({z\prime }_{i};\varphi_{2}\right))\right).$$

First, the autoencoder loss is a loss function designed to minimize the difference between the reconstructed features from the model and the original backbone features, while the discriminator loss serves to regularize the generated binary codes and continuous variables to resemble the target distribution. To update the autoencoder loss, the gradient is estimated using the Monte Carlo sampling method, which requires many iterations and results in prolonged training times.

To accelerate the convergence speed of the encoder’s training, we propose a new similarity loss function $L_{sim}$ that utilizes the backbone features. $L_{sim}$ generates an adjacency matrix by considering the correlations between features extracted through the backbone from the input data within a batch, then compares this with the binary bottleneck adjacency matrix of the model. By using the prior knowledge embedded in the backbone to create similarity labels for the adjacency matrix, which are then used in model training, we achieve faster convergence without significantly altering the existing model structure. Specifically, $L_{sim}$ follows these steps: First, the input data within a batch is passed through the backbone to extract features and, based on the correlations between these features, the adjacency matrix $A_{B}$ is computed.

(8) $$A_{B}^{i,j}=0.5\left(1+\frac{{x^{\prime }}_{i}\;\cdot \;{x^{\prime }}_{j}}{{\left\|{x^{\prime }}_{i}\right\|}_{2}\cdot {\left\|{x^{\prime }}_{j}\right\|}_{2}}\right),\;A_{bbn}^{i,j}=0.5\left(1+\frac{{b^{\prime }}_{i}\;\cdot \;{b^{\prime }}_{j}}{{\left\|{b^{\prime }}_{i}\right\|}_{2}\cdot {\left\|{b^{\prime }}_{j}\right\|}_{2}}\right)\;for\;i,\;j=1,2,\;\ldots ,\;B.$$

At this point, the adjacency matrix $A_{bbn}$ between the binary codes in the binary bottleneck structure is also calculated in the same manner.

(9) $$L_{sim}=\frac{1}{B^{2}}\sum_{i=1}^{B}\sum_{J=1}^{B}{\left(A_{bbn}^{i,j}-A_{B}^{i,j}\right)}^{2}.$$

By making the adjacency matrix of the backbone features similar to the adjacency matrix of the binary bottleneck through $L_{sim}$, the model inherits prior knowledge from the backbone, thereby improving the training speed and stability of the model. The total loss function of the proposed model, including the original TBH losses, is as follows.

(10) $$L_{TBH}=L_{AE}+L_{D}+L_{sim}.$$

The proposed system uses the improved model to pre-build the binary information of copyrighted images in the database. When a user inputs a query image, the system compares the binary feature vector of the query image with those stored in the database and recommends similar images. The comparison between vectors is based on Hamming distance, and the Top N images with the shortest Hamming distances are returned as the final retrieval results.

#### 3.3.2. Binary Code Group-Based Search

**Figure 4:**
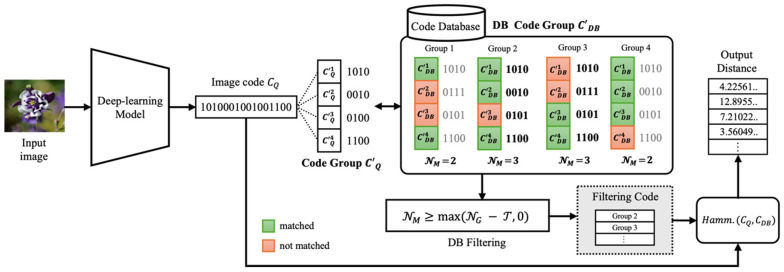
Code group-based image search process.

Figure 4 illustrates the vector search method, which improves search processing speed by efficiently calculating Hamming distances within the database. The query image $I_{Q}$ is converted into a binary feature vector $C_{Q}$ through the deep learning model, and in this study, the length of the binary vector $C_{bit}$ is set to 16 bits. The binary vector is then divided into four code groups, each of which is compared with the binary vectors in the database to maximize search efficiency. To search for images where the Hamming distance between binary codes is below the threshold T, the number of matching groups $N_{M}$ is calculated through code group comparisons, and images that satisfy $N_{M}\geq max(N_{G}-T,0)$ are filtered to enhance search speed. Finally, the Hamming distance is calculated only for the filtered images to generate the final list of similar images. Algorithm 1 shows the detailed operation process of the speed enhancement algorithm based on code groups.

This approach was first introduced by [21] and allows efficient Hamming distance calculation in large-scale databases by using a simple method of reducing the candidate set through group matching before calculating the Hamming distance. This significantly improves the database search speed in the copyright image infringement detection and retrieval process.
**Algorithm 1** Efficient hamming distance search from database pipeline**Input:** Query image $I_{Q}$ **Output:** Output Hamming distance list **Require:**
$\;N_{G},\;\;S_{G},T$    
 // Code group number, size, threshold
$$T^{\prime }=max(N_{G}-T,\;0)$$// Get binary code using a deep learning model and slice binary code to make code group$$C_{Q}=Model(I_{Q})$$$${C^{\prime }}_{Q}^{1},{C^{\prime }}_{Q}^{2},\;\ldots ,{C^{\prime }}_{Q}^{N_{G}}=Model(C_{Q},N_{G},\;S_{G}\;)$$$//\;C_{Q}$ $=1101001110101111\;\mathrm{And}\;N_{G},\;S_{G}$ = 4 $//\;{C^{\prime }}_{Q}^{1}$ $=1101,\;{C^{\prime }}_{Q}^{2}$ $=0011,\;{C^{\prime }}_{Q}^{3}$ $=1010,\;{C^{\prime }}_{Q}^{4}$ = 1111 // Execute the query and save the query results in
R SELECT * (
  CASE WHEN = THEN 1 ELSE 0 END +
  CASE WHEN = THEN 1 ELSE 0 END +
  ...  
CASE WHEN = THEN 1 ELSE 0 END ) AS FROM DATABASE WHERE (  ${C^{\prime }}_{DB}^{1}$ $={C^{\prime }}_{Q}^{1}\;$ OR  ${C^{\prime }}_{DB}^{2}$ $={C^{\prime }}_{Q}^{2}\;$ OR  
…  ${C^{\prime }}_{DB}^{N_{G}}$ $={C^{\prime }}_{Q}^{N_{G}}$ $)\;\mathrm{HAVING}\;N_{M}\geq T$ // Calculate the Hamming distance **for in do** $\;\;\;\mathrm{Calculate}\;HammingDist(C_{Q},\;C_{DB}$)   And filtering Hamming dist ≤ T  **end for** 

## 4. Experiments

### 4.1. Implementation Details

The proposed system was implemented using different deep learning frameworks for each module. The CVAE model in the infringement detection module was implemented with TensorFlow 2.8.0, and the TBH model for image retrieval was implemented with PyTorch 2.0.1. The entire system runs on an Ubuntu 20.04 environment equipped with a single NVIDIA RTX A6000 with 48 GB of memory. The system uses two Intel Xeon Silver 4310 CPUs, each with 12 cores, and runs at a base clock speed of 2.10 GHz, with 512 GB of RAM. For training the copyright infringement detection model, the batch size was set to 512, and a cosine scheduler was applied with a learning rate of 0.0001 for 1000 epochs. Each epoch took approximately 9 min and 26 s. The search thresholds for the infringement detection module were set to $T_{1st}$ and $T_{2nd}$, with values of 12 and 0.9, respectively. The image retrieval model utilized the TBH architecture, with a batch size of 128 set during the training process. The network was optimized using the Adam optimizer with a learning rate of 0.0001, and the learning rate was decreased using a cosine decay scheduler. The dimensionality of the feature size input to the model was set to 768, and training was conducted for up to 500 epochs, with early stopping applied to prevent overfitting.

### 4.2. Datasets

Flickr25k is a dataset consisting of 25,000 images, each with an average of 8.94 tags, and is one of the mainstream benchmarks used in image retrieval. For training the image retrieval model, we used 5000 images for training, 2000 images for testing, and the remaining images as the database dataset.

CIFAR10 consists of 60,000 images across 10 classes, with a relatively small resolution of 32 × 32. From the total of 60,000 images, we first split off 10,000 images as the test data for the image retrieval model and then evaluated the model using two different methods. In CIFAR10-I, the remaining 50,000 images, excluding the test data, were used for both training and the database. In CIFAR10-II, 5000 images were allocated for training, and the remainder were used as the database.

NUS-WIDE is a dataset containing 269,648 images, with 81 concepts and 5018 tags. We used 195,834 images belonging to 20 concepts for training the image retrieval model, and the remaining images, excluding 2100 pieces of test data, were used for both training and the database.

KOLAA Copyright Image [22] is a private image dataset provided by the Korea Literary and Artistic Copyright Association, consisting of approximately 380,000 images with about 7 to 11 labels per image. We used 370,000 images for training and 1000 images for testing the copyright infringement detection model. For the image retrieval model, 5000 images were randomly selected for training, 2000 images for testing, and 10,000 images as the database dataset.

### 4.3. Evaluation of Hashing Model

The performance of the proposed method was compared with that of existing hashing models on various benchmark sets (Flickr25k, CIFAR10, NUS-WIDE) and the KOLAA copyright image dataset. Performance evaluation was conducted using the mean Average Precision (mAP) metric. The mAP was calculated for the top k results with the highest similarity. The values of k were set to 5000 for Flickr25k and NUSWIDE, 1000 for CIFAR10, and 500 for KOLAA, with all datasets evaluated by generating hash codes of 16-, 32-, and 64-bit lengths. We compared the performance of Bi-halfNet [23], CIBHash [24], DSH [25], the existing TBH model, and the proposed model.

As shown in Table 1, the improved image retrieval model outperformed the existing TBH model across all datasets and achieved relatively higher results compared to other hashing models. On the CIFAR10-1 dataset, the proposed model demonstrated exceptional performance with a difference of up to 26%. Additionally, in the average model performance across all benchmarks, the proposed model achieved the highest mAP result of 78.9%, followed by CIBHash (70.8%), Bi-HalfNet (66.5%), TBH (65.8%), and DSH (64.1%). The experimental results demonstrate that the proposed model can generate unique hash codes that effectively represent images by leveraging the self-attention mechanism of the ViT backbone, which accurately identifies key features within images.

**Figure 5:**
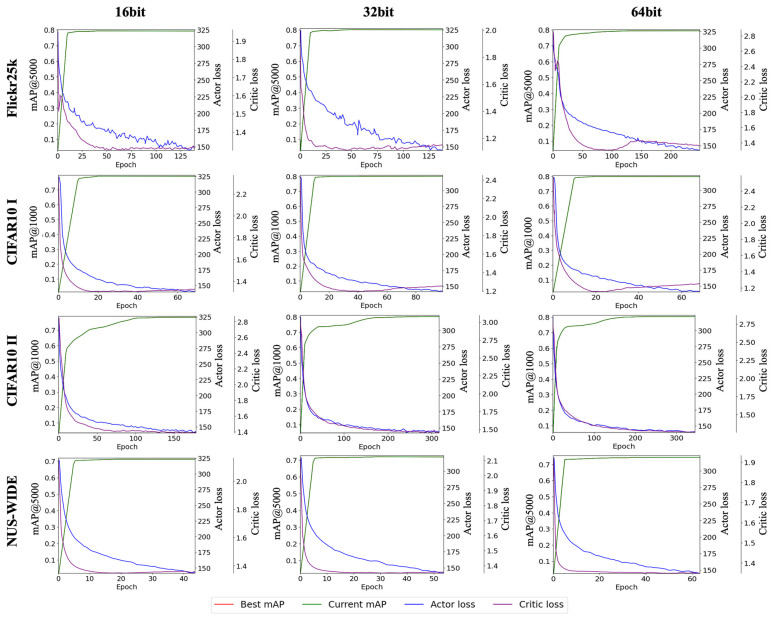
Training curves and mAP scores for different datasets.

Figure 5 illustrates the training process of the improved image retrieval model, showing the learning trends for 16, 32, and 64 bits on the Flickr25k, CIFAR10, and NUS-WIDE datasets. The best mAP, current mAP, actor loss, and critic loss are represented by red, green, blue, and purple lines, respectively. The graph shows that the current mAP curve closely aligns with the best mAP curve, indicating that the model is being trained appropriately. Notably, all graphs exhibit a sharp increase in performance during the early stages of training, suggesting that the model is effectively optimizing learning and accelerating convergence by leveraging diverse feature information. However, compared to the CIFAR10 learning trend, the loss curves for Flickr25k and NUS-WIDE show relatively slow and unstable declines. This could indicate that the model requires more time to learn diverse features or that the complexity of these datasets is relatively higher.

**Figure 6:**
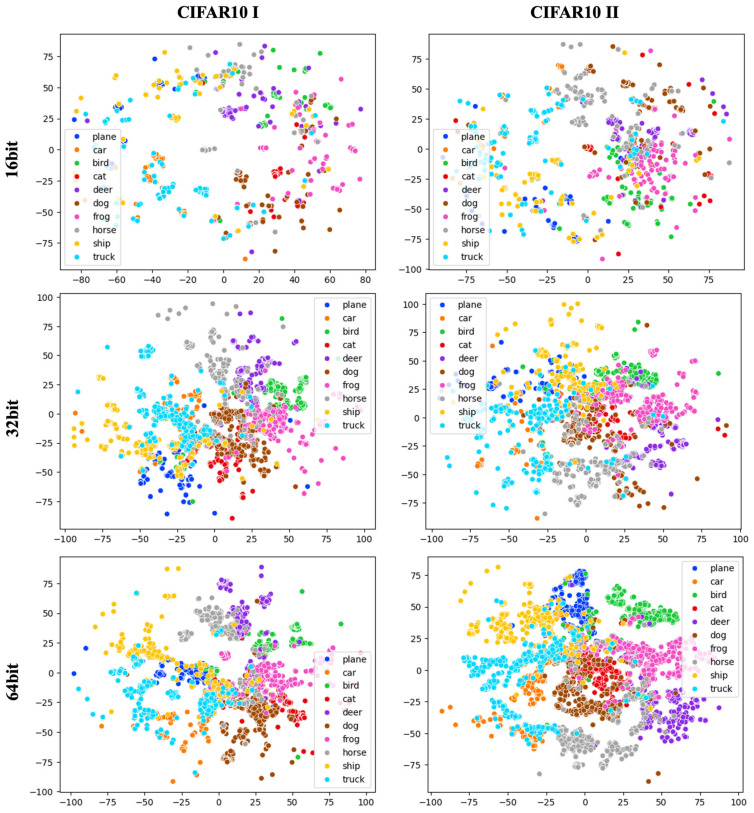
T-SNE visualization results for CIFAR10 I and CIFAR10 II.

**Figure A1:**
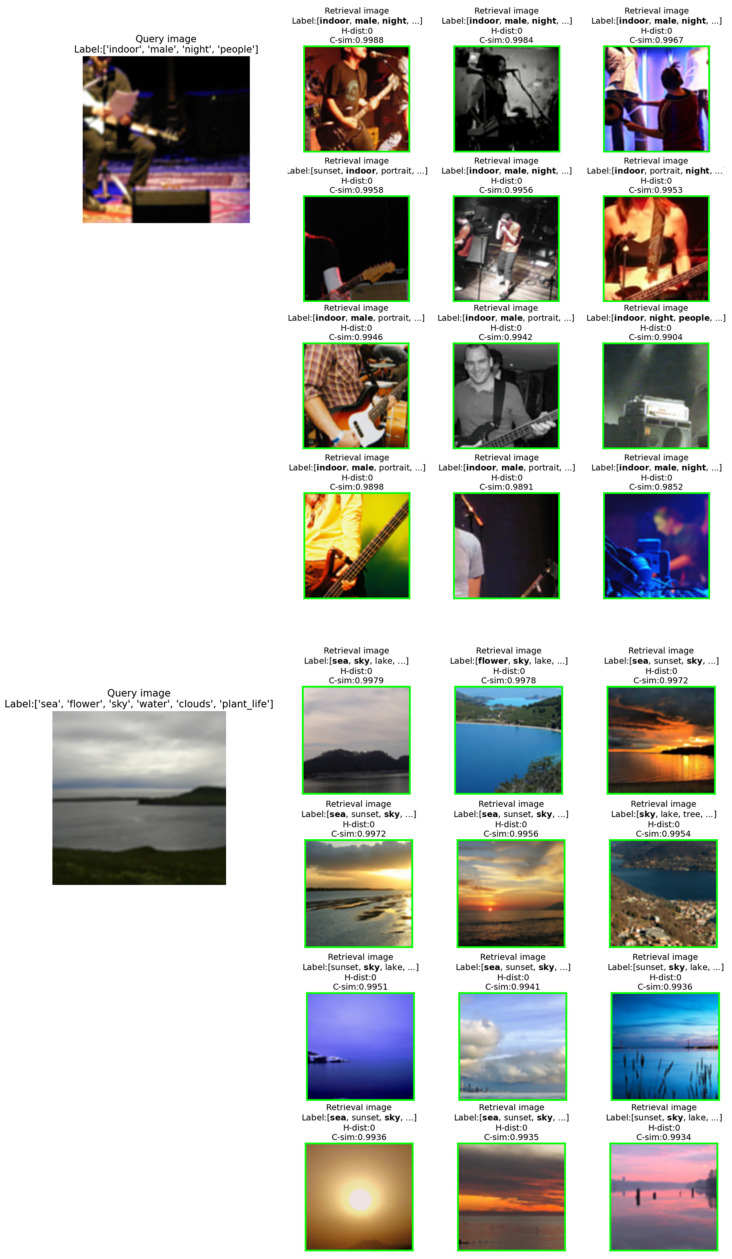
Detailed image retrieval results for Flickr25k.

**Figure A2:**
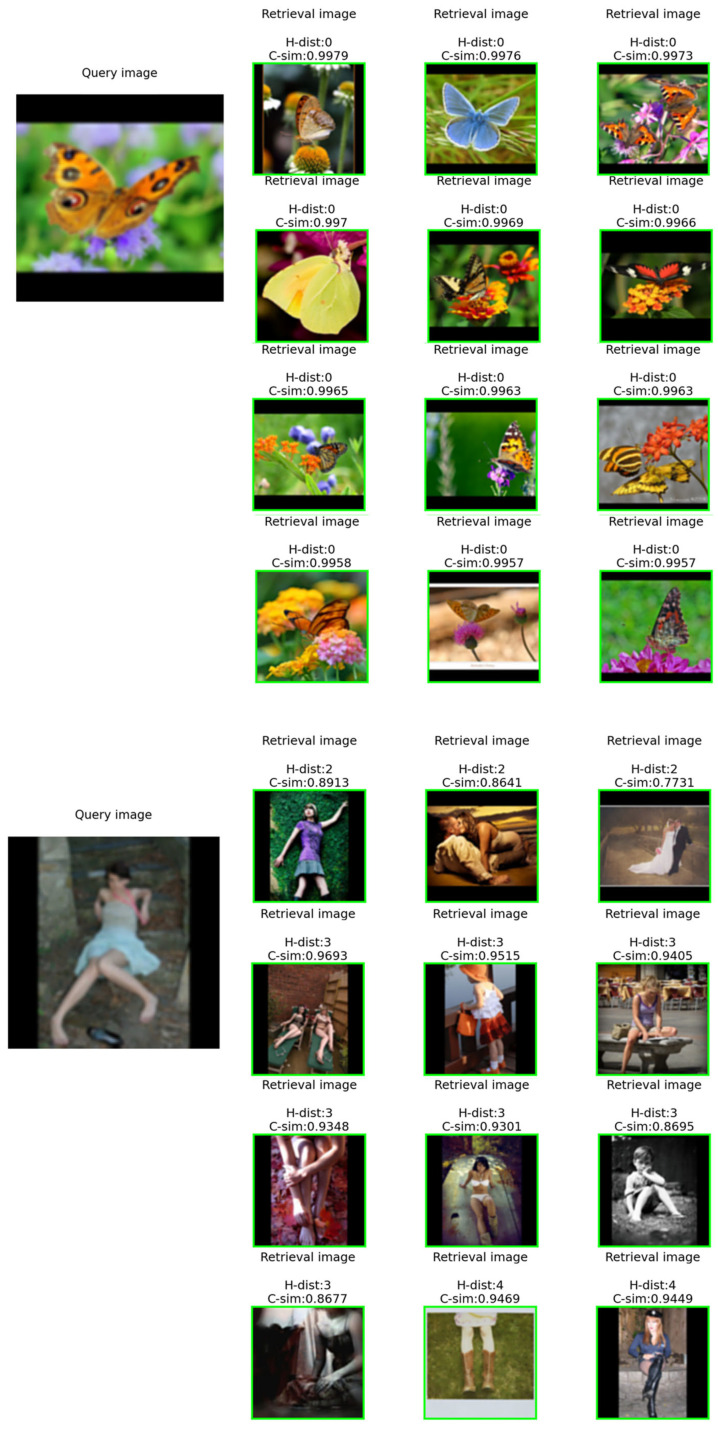
Detailed image retrieval results for NUS-WIDE.

**Figure A3:**
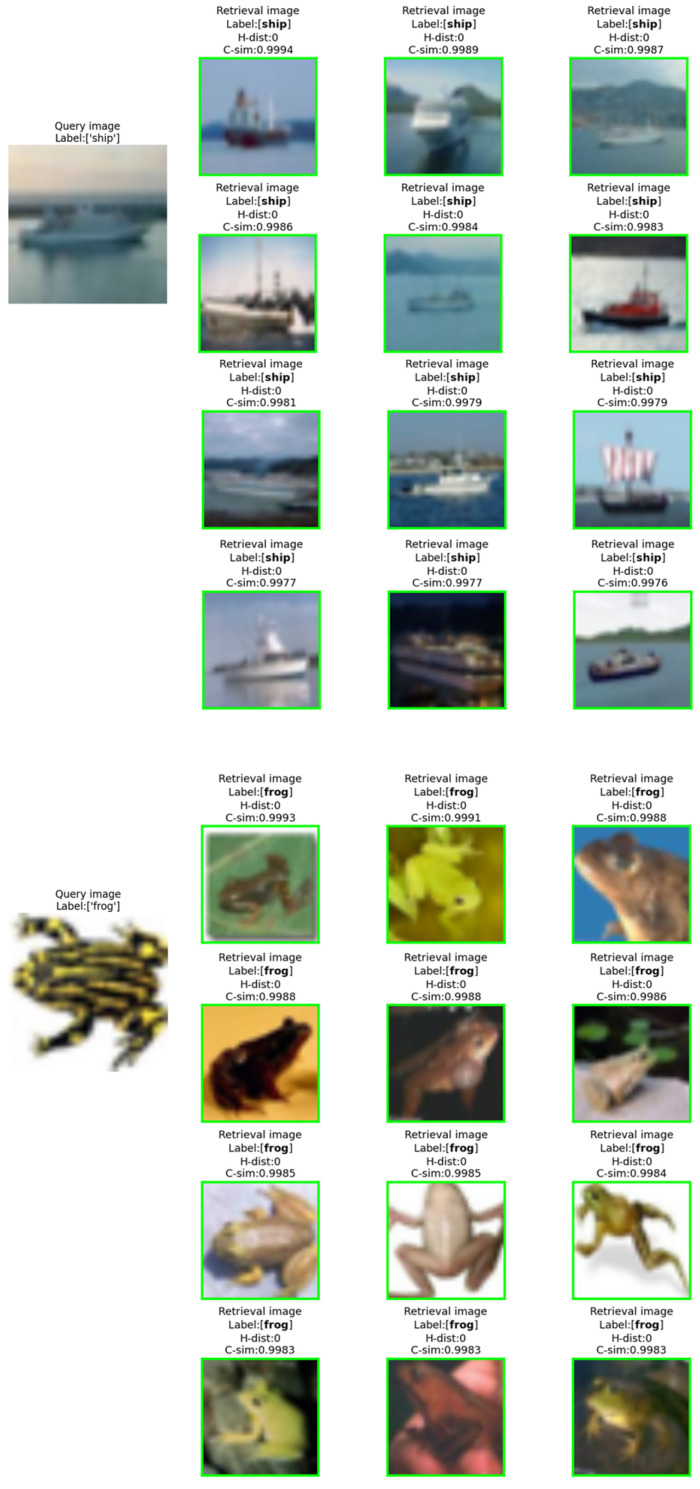
Detailed image retrieval results for CIFAR10 I.

**Figure A4:**
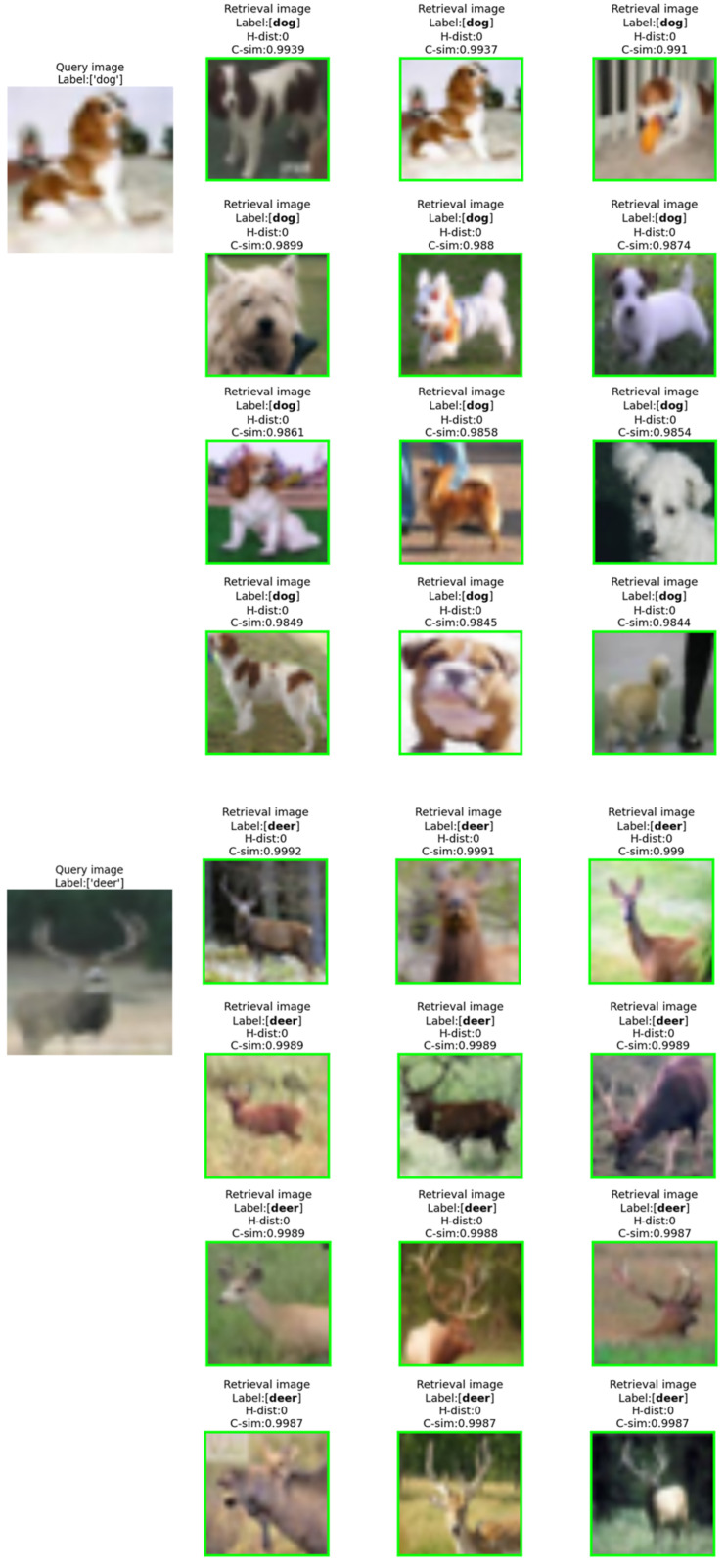
Detailed image retrieval results for CIFAR10 II.

**Figure A5:**
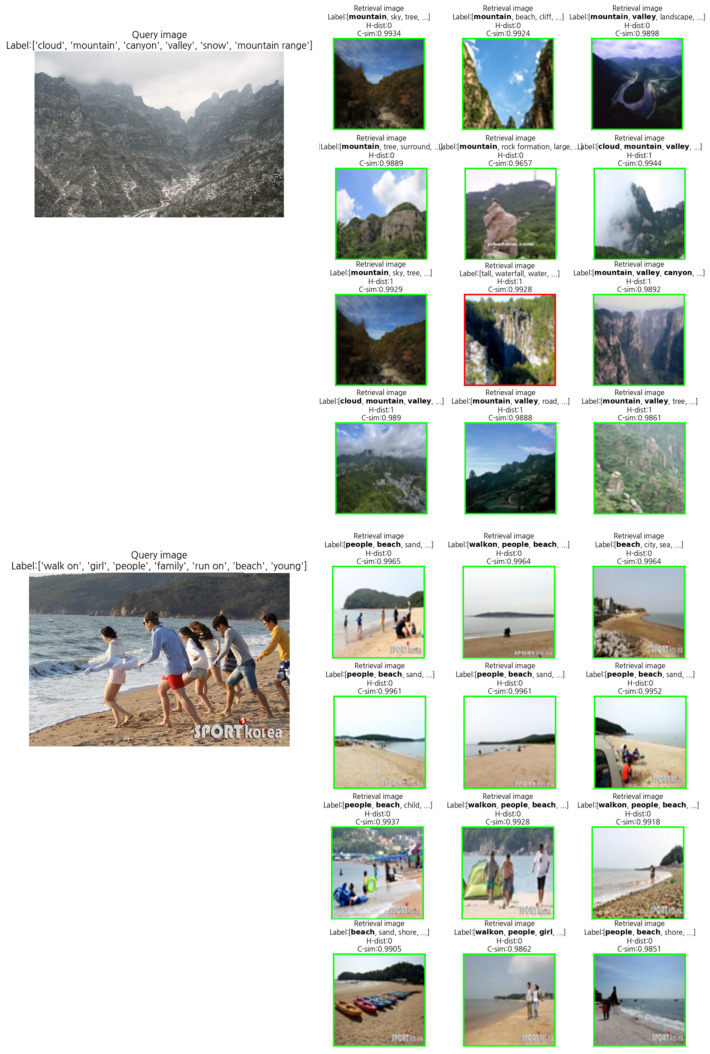
Detailed image retrieval results for KOLAA Image.

Figure 6 presents the experimental retrieval results of the image retrieval module, visualized using T-SNE. The dataset used in the experiment is the two CIFAR10 datasets mentioned in Section 4.2, representing the distribution of 10 classes. In the 16-bit results, the clusters appear somewhat regionally localized, but as the bit length increases to 32 and 64, the distances between clusters decrease, leading to more cohesive groupings. Larger bit lengths reduce the likelihood of overlapping Hamming distances due to the longer vector length, thus improving accuracy. However, there is a trade-off with increased memory usage and computational costs. Additionally, when comparing the two datasets, CIFAR10 II, which was trained with fewer training data and with no overlap between the training and database sets, shows more compact clustering of each class compared to CIFAR10 I. This can be interpreted as an indication that the model improves performance by effectively handling diverse data without overfitting to specific data. In particular, the good performance on untrained data suggests the potential for zero-shot learning. Additionally, the Appendix A provides practical examples of the proposed approach through Figure A1, Figure A2, Figure A3, Figure A4 and Figure A5.

### 4.4. User Satisfaction Analysis

To validate the effectiveness and practicality of the proposed copyright-free image recommendation system, a user satisfaction analysis was conducted. The focus was particularly on verifying whether teachers who create educational content could effectively prevent copyright infringement and receive efficient image retrieval. Among the 65 participating teachers, 47% were elementary school teachers, 31% were middle school teachers, and 22% were high school teachers. Out of the 380,000 copyrighted images owned by KOLAA, 180,000 images were used to build the infringement detection DB and retrieval DB through the system’s modules.

**Figure 7:**
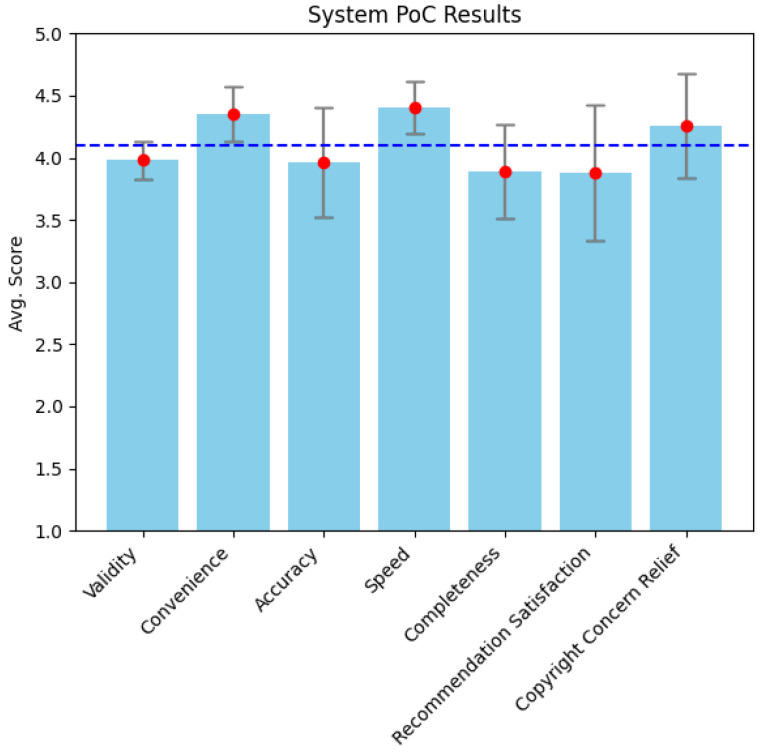
User satisfaction results of copyright-free image recommendation system.

Teachers used the system to search for images related to their teaching materials and to verify copyright inspection results. During this process, a total of 1757 retrieval results were generated, and reviews were written for each retrieval. Teachers evaluated their overall experience and satisfaction with the system using 13 evaluation items. The main evaluation metrics included Validity, Convenience, Accuracy, Speed, Completeness, Retrieval Satisfaction, and Copyright Concern Relief, which are graphically represented in Figure 7.

The analysis of the data collected using the Likert scale revealed that the overall satisfaction with the system was an average of 3.88 points (standard deviation of 0.55), indicating that users generally evaluated the system positively. The evaluation score for solving copyright image issues was particularly high, with an average of 4.26 points (standard deviation of 0.42). Notably, the Convenience (average of 4.35 points) and Speed (average of 4.40 points) metrics showed very high satisfaction, indicating that teachers experienced a high level of satisfaction when using the system.

## 5. Conclusions

This study designed a copyright-free image recommendation system for educators that checks copyright images and recommends similar alternatives. The proposed system provides users with image infringement detection and image retrieval functions through two modules. Notably, the system’s performance was enhanced over existing retrieval models by modifying the backbone network of the image retrieval model and introducing a new loss function, while also improving processing speed through group code matching.

Considering the results of user satisfaction, the positive aspects of the system proposed in this study can be summarized as follows. Teachers highly rated the system as an effective tool for addressing copyright issues. Notably, the high scores for alleviating concerns about copyright images indicate that the system significantly contributed to reducing legal issues in the creation of educational materials. This allows teachers to confidently use images when developing educational content, thereby providing students with a wider array of visual resources and enhancing the quality of education.

These results demonstrate that the proposed system performed exceptionally well in detecting copyright infringement and retrieving images. Additionally, the high evaluation of speed is attributed to the efficient design of the hash code within the system. This design enhances data search and processing speed, optimizing system performance and leading to high ratings for both accuracy and speed. However, the large standard deviation in the accuracy and speed metrics suggests that further optimization is needed to ensure consistent performance across diverse user environments. Given the flexibility and scalability of the hash code design, there is ample room for future improvement. Also, the potential of zero-shot learning was partially observed in image hashing model research, indicating that further studies are needed to examine its effectiveness in more diverse and practical datasets.

Based on these findings, future research will focus on applying various deep learning models. To further improve the system’s performance, there is a need to collect and test a wider variety of copyright image datasets. Additionally, incorporating a user-centric feedback learning mechanism could allow the system to progressively learn and improve based on feedback from educators, leading to more tailored search results.

## Figures and Tables

**Table 1:** Comparison with other hashing models.

Dataset	Bits	Hashing Model				
		TBH [12]	Bi-halfNet [23]	CIBHash [24]	DSH [25]	Ours
Flickr25k [3] mAP@5000	16	0.743	0.760	0.772	0.677	0.802
	32	0.761	0.779	0.784	0.679	0.809
	64	0.778	0.786	0.795	0.712	0.812
CIFAR10 I [4] mAP@1000	16	0.546	0.561	0.593	0.651	0.760
	32	0.586	0.576	0.636	0.661	0.774
	64	0.624	0.595	0.651	0.676	0.789
CIFAR10 II [4] mAP@1000	16	0.532	0.499	0.590	0.640	0.759
	32	0.573	0.520	0.622	0.652	0.766
	64	0.578	0.553	0.641	0.669	0.794
NUS-WIDE [5] mAP@5000	16	0.717	0.768	0.790	0.552	0.794
	32	0.725	0.783	0.807	0.558	0.804
	64	0.735	0.799	0.815	0.562	0.810
KOLAA Copyright [22] mAP@500	16	0.544	0.614	0.633	0.598	0.650
	32	0.619	0.644	0.657	0.601	0.711
	64	0.629	0.667	0.673	0.623	0.715

## Data Availability

The original data presented in the study are openly available in the CIFAR-10 dataset at https://www.cs.toronto.edu/~kriz/cifar.html (accessed on 8 April 2009), the NUS-WIDE dataset at https://dl.acm.org/doi/10.1145/1646396.1646452 (accessed on 8 July 2009), and the Flickr25K dataset at https://press.liacs.nl/mirflickr/ (31 October 2008). The KOLAA image datasets presented in this article are not readily available because the data are part of an ongoing study. Requests to access the datasets should be directed to KOLAA.

## Appendix A

This section provides additional visual results from the copyright-free image recommendation system discussed in the main paper. The leftmost large image is the input query image, while the smaller images on the right represent the search results. The similarity decreases from left to right in the first row and from left to right in the second row. The first image of the second row follows the last image of the first row in terms of similarity ranking. Images that share the same tag as the query image are highlighted with a green border, while those that do not share the tag are outlined in red. The original resolution of the CIFAR10 dataset was 32 × 32, then we enlarged all images for display.
